# Translation, Cultural Adaptation, and Evaluation of a Brazilian Portuguese Questionnaire to Estimate the Self-Reported Prevalence of Gluten-Related Disorders and Adherence to Gluten-Free Diet

**DOI:** 10.3390/medicina55090593

**Published:** 2019-09-15

**Authors:** Jesús Gilberto Arámburo-Gálvez, Itallo Carvalho Gomes, Tatiane Geralda André, Carlos Eduardo Beltrán-Cárdenas, María Auxiliadora Macêdo-Callou, Élida Mara Braga Rocha, Elaine Aparecida Mye-Takamatu-Watanabe, Vivian Rahmeier-Fietz, Oscar Gerardo Figueroa-Salcido, Feliznando Isidro Cárdenas-Torres, Noé Ontiveros, Francisco Cabrera-Chávez

**Affiliations:** 1 Unidad Academica de Ciencias de la Nutrición y Gastronomia, Universidad Autónoma de Sinaloa, Culiacán, Sinaloa 80019, Mexico; gilberto.aramburo.g@gmail.com (J.G.A.-G.); carlos.1.beltran@hotmail.com (C.E.B.-C.); : feliznandoc@hotmail.com (F.I.C.-T.); 2 Posgrado en Ciencias de la Salud, División de Ciencias Biológicas y de la Salud, Universidad de Sonora, Hermosillo, Sonora 83000, Mexico; 3 Programa de Maestría en Ciencias en Enfermeria, Facultad de Enfermería, Los Mochis, Sinaloa 81220, Mexicotatianegrandre@gmail.com (T.G.A.); 4 Faculdade de Juazeiro do Norte, Juazeiro do Norte, Ceará 63010-215, Brazil; auxiliadora.callou@fjn.edu.br (M.A.M.-C.); elidamara@usp.br (É.M.B.R.); 5 Universidade Estadual de Mato Grosso do Sul, Dourados, Mato Grosso do Sul 79804-970, Brazil; swatanab@terra.com.br (E.A.M.-T.-W.); vivian@uems.br (V.R.-F.); 6 Division of Sciences and Engineering, Department of Chemical, Biological, and Agricultural Sciences (DC-QB), Clinical and Research Laboratory (LACIUS, URS), University of Sonora, Navojoa 85880, Sonora, Mexico

**Keywords:** celiac disease, gluten-free diet, gluten-related disorders, NCGS, self-report, survey studies

## Abstract

*Background*: A Spanish version of a questionnaire intended to estimate, at the population level, the prevalence rates of self-reported gluten-related disorders and adherence to gluten-free diets has been applied in four Latin American countries. However, idiom issues have hampered the questionnaire application in the Brazilian population. Thus, the aim of the present study was to carry out a translation, cultural adaptation, and evaluation of a Brazilian Portuguese questionnaire to estimate the self-reported prevalence of gluten-related disorders and adherence to gluten-free diets in a Brazilian population. *Materials and Methods*: Two bilingual Portuguese–Spanish health professionals carried out the translation of the original Spanish version of the questionnaire to Brazilian-Portuguese. Matching between the two translations was evaluated using the WCopyFind.4.1.5 software. Words in conflict were conciliated, and the conciliated version of the Brazilian Portuguese instrument was evaluated to determine its clarity, comprehension, and consistency. A pilot study was carried out using an online platform. *Results*: The two questionnaires translated into Brazilian Portuguese were highly matched (81.8%–84.1%). The questions of the conciliated questionnaire were clear and comprehensible with a high agreement among the evaluators (*n* = 64) (average Kendall’s W score was 0.875). The participants did not suggest re-wording of questions. The answers to the questions were consistent after two applications of the questionnaire (Cohen’s k = 0.869). The pilot online survey yielded low response rates (9.0%) highlighting the need for face-to-face interviews. *Conclusions*: The translation and evaluation of a Brazilian Portuguese questionnaire to estimate the self-reported prevalence rates of gluten-related disorders and adherence to gluten-free diets was carried out. The instrument is clear, comprehensible, and generates reproducible results in the target population. Further survey studies involving face-to-face interviews are warranted.

## 1. Introduction

The spectrum of gluten-related disorders (GRD) involves celiac disease (CD), wheat allergy and non-celiac gluten sensitivity (NCGS). Patients under this spectrum should follow a gluten-free diet (GFD) to avoid the gastrointestinal and/or extraintestinal symptoms triggered by gluten. In fact, survey studies have proven that following a GFD can improve the health-related quality of life in CD patients in spite of the difficulties of following the diet [1]. Different from wheat allergy and NCGS, untreated CD could affect the nutritional status and predispose to other conditions such as osteoporosis [2,3], anemia [4], and intestinal T-cell lymphoma [5]. On its own, following a GFD without medical/dietitian advice can predispose not only to deficiencies in micronutrients, but also to low fiber intake [6,7] increasing the risk of dyslipidemia [8,9]. Notably, recent survey-based studies carried out in Latin American countries have shown that both CD and NCGS are largely underdiagnosed in Mexico, Colombia, and El Salvador, and that most people following a GFD are doing it for reasons other than health related benefits, as well as without medical advice [10,11,12]. This is not the case in Argentina, a country that has implemented programs for the detection of CD and for ameliorating the economic burden of following a GFD [13]. The questionnaire utilized in these studies is a Spanish version, and this has hampered its application in the Brazilian population. Recently, an Italian instrument [14] designed to estimate the prevalence of NCGS in clinical settings has been translated to Brazilian Portuguese [15], but a validated questionnaire intended to evaluate, at population level, the self-reported prevalence of GRD and adherence to a GFD in Brazilians is not available yet. Thus, as part of an attempt to expose the magnitude and relevance of the underdiagnosis of GRD and the adherence to GFD in the Latin American region, the aim of the present work is to generate and test a Brazilian Portuguese version of a validated Spanish questionnaire designed to estimate the self-reported prevalence of GRD and adherence to a GFD.

## 2. Materials and Methods

### 2.1. Questionnaire

The questionnaire is based on a previously designed and tested instrument that is utilized in Spanish-speaking populations [10,11,12,13]. The questionnaire includes 2 sections. The first section was designed for those who report adverse reactions after wheat/gluten ingestion, and the second one for those who do not report them. The participants should answer questions related to the symptoms triggered after gluten ingestion, time of appearance of the symptoms, adherence to a GFD or gluten avoidance and the motivations of doing so, among other questions (Supplementary Material Section S1).

### 2.2. Translation and Back-Translation

The translation process of the questionnaire was carried out as previously described, with minor changes [16,17]. The procedure was as follows: two health professionals, Portuguese–Spanish bilingual, but also Brazilian Portuguese native speakers, realized the translation of the questionnaire from Spanish to Brazilian Portuguese (TBP1 and TBP2). The matches between translations were analyzed using the WCopyFind.4.1.5 software (Charlottesville, VA, USA) to determine literal match by words (ignoring phrases, all punctuations, outer punctuations, numbers, letter case, and skipping non-words, selecting Brazilian-Portuguese as the base language). After conciliation of the words in conflict (words that did not match) by the Spanish-Portuguese translators, a conciliate version of the questionnaire was elaborated and back-translated to Spanish by two Spanish-Portuguese bilingual professionals who were also Spanish native speakers. The match between the back-translated questionnaires (from Brazilian Portuguese to Spanish; two versions) and the match between each back-translated version with the original Spanish version of the questionnaire were evaluated as previously described (selecting Mexican Spanish as the base language). All matches were reported as percentage.

### 2.3. Questionnaire Clarity, Comprehension and Wording of Questions Evaluation

Clarity and comprehension of the conciliated questionnaire in Brazilian Portuguese was evaluated as previously described [10,16]. A digital version of the Brazilian Portuguese questionnaire was constructed using the SurveyMonkey platform (San Mateo, CA, USA). Brazilian Portuguese native speakers (*n* = 64) received a text message with the link to the questionnaire. Afterwards, participants proceeded to evaluate the clarity and comprehension of all the questions.

The evaluation was initially performed using a numerical scale from 0 to 10 (0 = very easy to understand; 10 = very difficult to understand). Questions rated with values ≤3 were considered as clear and comprehensible, therefore, rewording was not required [10]. Results were reported with 95% confidence intervals. Furthermore, clarity/comprehension was evaluated using a cognitive survey that evaluated each item/question in a three-point ordinal scale; 1: Clear and comprehensible, 2: Difficult to understand, and 3: Incomprehensible [16]. Agreement among participants was evaluated using the Kendall’s W coefficient of concordance, ranging from 0 (no agreement) to 1 (complete agreement). A W value ≥ 0.66 was considered as an adequate agreement among the participants. Additionally, to ensure the comprehension of each question, the participants were to answer the following question: In case you do not understand the question, how would you write it? This option was provided if the participants did not correctly understand some questions, or if they thought that there was a more comprehensible way to write the item.

### 2.4. Questionnaire Test-Retest Consistency

The questionnaire reproducibility was evaluated in a cohort of subjects who reported adverse reactions to wheat/gluten (*n* = 12), as well as in another cohort who reported adverse reactions to foods other than gluten (*n* = 8). Participants answered the questionnaire twice. The time period interval between the first and second application of the questionnaire was at least one week. The reproducibility of the questionnaire was evaluated with Cohen’s *k* coefficient tests.

### 2.5. Pilot Survey

After the clarity/comprehension and consistency evaluation process, a digital version of the conciliated Brazilian-Portuguese questionnaire (Supplementary Material Section S2) was sent to 966 Brazilian health sciences students from Faculdade do Juazeiro do Norte in Juazeiro do Norte, Ceará, Brazil using the SurveyMonkey platform (San Mateo, CA, USA). The first page of the survey showed a general description of the project and presented the consent form. All data were collected in June 2019. Inclusion criteria were as follows: subjects must be aged ≥ 18 years old, and able to read and answer the questionnaire by themselves. Exclusion criteria were as follows: subjects being < 18 years old or not being able to complete the questionnaire by themselves. Individuals were classified according to previously published definitions on GRD [12] (Supplementary Material Section S3).

### 2.6. Statistical Analysis and Ethical Issues

Statistical analysis was carried out using PASW statistics version 25.0 (SPSS Inc., Chicago, IL, USA). Total numbers, percentages, and 95% confidence intervals (CI) were analyzed according to a set of descriptive statistics. A *p* value < 0.05 was considered as statistically significant. OpenEpi software version 3.03a (Atlanta, GA, USA) was used to estimate the prevalence rates (95% CI) per 100 inhabitants. This study was approved by the Research Ethics Committee of the Faculdade do Juazeiro do norte (Número do Parecer: 3.382.689).

## 3. Results

### 3.1. Questionnaire Translation and Back-Translation

**Figure 1:**
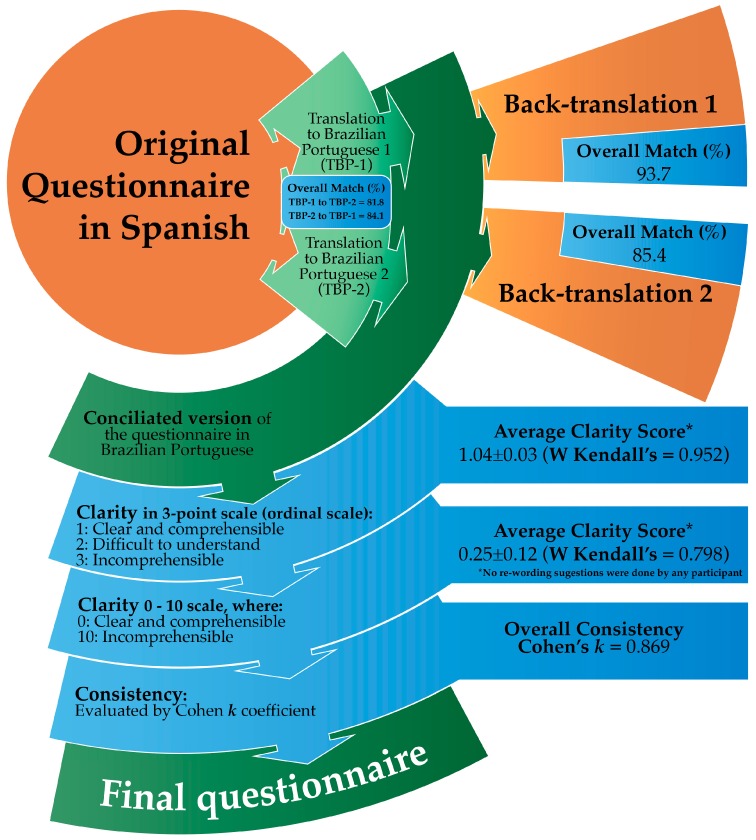
Flow chart of the translation of the questionnaire and the results of the evaluations on matches (between translations 1 and 2 from Spanish to Brazilian Portuguese, and between back-translations to Spanish compared to the original questionnaire), clarity, comprehension, and consistency.

The complete flow chart and the results of the evaluation of translation, clarity, comprehensibility, and consistency of the questionnaire are shown in Figure 1. Two native Brazilian Portuguese speakers carried out the translations from Spanish to Brazilian Portuguese. Translations to Brazilian-Portuguese (TBP1 and 2) had more than 80% of an overall match between them (TBP1 matched 81.8% with TBP2 and TBP2 matched 84.1% with TBP1). Most of the items in conflict were synonymous in Brazilian Portuguese with the same meaning in Spanish language. After agreement by the translators, the best synonymous were selected to have a conciliated version of the questionnaire translated to Brazilian Portuguese. The back-translations (two versions) of the conciliated Brazilian Portuguese version of the instrument matched 93.7% and 85.4% with the original Spanish version.

### 3.2. Questionnaire Clarity/Comprehension

Sixty-four Brazilian Portuguese native speakers (38 females, 26 males; 18–55 years old) evaluated clarity/comprehensibility using a continuous scale (0: clear and comprehensible, 10: incomprehensible). On the bases of this evaluation, the average of the clarity score was 0.25 (CI, 95%: 0.03–0.53; values ranged from 0 to 9) and the Kendall’s W score was 0.798. Using the three-point ordinal scale, the average of the clarity score was 1.04. This value is very close to “clear and comprehensible” and, according to the Kendall’s W score obtained (0.952), involves a high concordance among the individuals’ answers to the questions. Importantly, when the participants were asked for the questions’ re-writing to improve the understanding, neither re-wording nor suggestions for changes were reported.

### 3.3. Questionnaire Consistency

Twenty participants who reported adverse reactions to gluten or to other foods answered the questionnaire twice (12 females and 8 males). The concordance between the first and the second application of the questionnaire was measured individually, and the average of Cohen’s *k* coefficient was 0.869 (Figure 1). This *k* value can be interpreted as an almost perfect concordance.

### 3.4. Pilot Study

A total of 966 health sciences students received the link to answer the questionnaire. The response rate was 9.0% (*n* = 87), but 13 subjects had to be excluded due to their proportioned incomplete demographic data or responses. Thus, a total of 74 valid questionnaires were considered for prevalence estimations. The proportion of male/female was 24.3%: 75.6% (male: 18; female: 56). Average age was 25 ± 6.6 years. The most commonly self-reported, physician-diagnosed conditions were psychiatric diseases (9.45%; IC 95%), irritable bowel syndrome (8.1%; IC 95% 3.88–18.52), diabetes, lactose intolerance, and allergies (6.75%; IC 2.23–15.07, each). However, due to the reduced number of participants, risk analysis between Self-Reported Gluten Sensitivity (SR-GS) and non-Self-Reported Gluten Sensitivity (non-SR-GS) conditions could not be calculated.

Prevalence rates estimations of GRD and other adverse foods reactions are shown in the Table 1. Adverse reactions to wheat/gluten were reported by 16.21% of the participants, though, only two fulfilled criteria for SR-GS (2.70%) (Supplementary Material Section S3). The prevalence rates of wheat allergy and NCGS were 1.35% each. No male fulfilled the criteria for either wheat allergy or NCGS. Physician diagnosis of CD was not reported in this pilot study. The prevalence rate of adherence to a GFD was higher in females than in males, while the prevalence rate of wheat/gluten avoiders was slightly higher in males than in females (*p* > 0.05).

**Figure 2:**
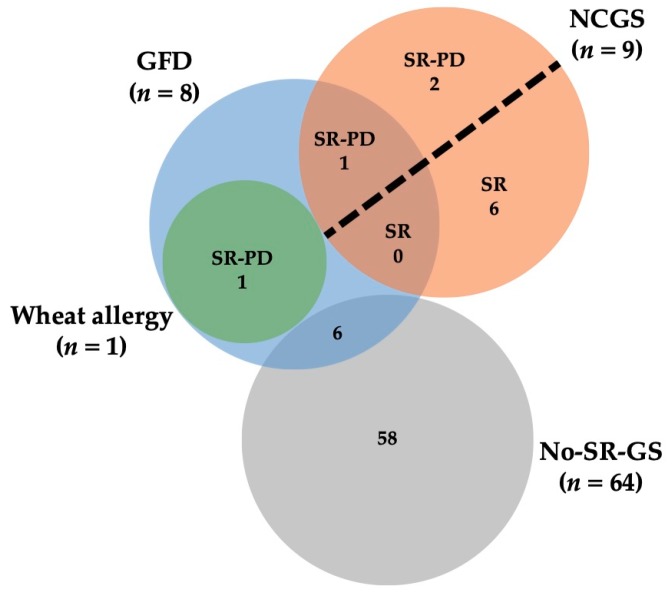
Characteristics of the participants who were following a GFD. SR-PD: Self-reported Physician-Diagnosed; SR: Self-reported; GFD: Gluten-free Diet; NCGS: Non-Celiac Gluten Sensitivity; Non-SR-GS: Non-self-reported Gluten Sensitivity.

The characteristics of the individuals following a GFD are shown in Figure 2. It should be noted that almost all participants who were following a GFD (75%) and those that were avoiding wheat/gluten containing foods (75%) fulfilled criteria for non-SR-GS. Regarding the motivations for following a GFD, in the non-SR-GS group the most frequent motivation was weight control (50%), while in the SR-GS group was the symptomatic relapse. Similar results were obtained in the wheat/gluten avoiders group. All participants who were following a GFD reported to be under the supervision of a dietitian to follow the diet. Ten individuals reported recurrent gastrointestinal and/or extra-intestinal symptoms triggered after the ingestion of wheat/gluten containing foods. Bloating (80%), abdominal pain (80%), nausea (60%), stomachache (60%), and reflux (60%) were the most commonly reported gastrointestinal symptoms. On the other hand, lack of wellbeing (60%), tiredness (40%), and muscular pain (40%) were the most commonly reported extra-intestinal symptoms.

## 4. Discussion 

Survey-based studies are useful to estimate the prevalence rates of several conditions and set the ground for further epidemiological studies based on objective diagnostic criteria. The results of survey studies can be interpreted in different ways. Particular attention needed to be given to the most underdiagnosed conditions and those conditions for which there is a lack of sensitive and specific biomarkers, such as CD and NCGS, respectively. In this context, a questionnaire intended to evaluate the self-reported prevalence of GRD and the adherence to a GFD in Spanish-speaking populations was applied in four Latin American countries [10,11,12,13]. However, idiom issues hampered the application of this instrument in Brazilian Portuguese speakers, the largest population in South America. To fill this gap, the questionnaire was translated to Brazilian Portuguese and systematically tested. The translations of the questionnaire from Spanish to Brazilian Portuguese matched in high percentage and most of the items in conflict were words with the same meaning. The similarity between Spanish and Portuguese is the highest among romance languages [18], thus allowing for the facilitation of the translation of the questionnaire and, at the same time, it could improve the matching among the translations carried out by different translators. Back-translation is a process necessary to verify the accuracy of the original translation [19] and to reduce any discrepancies between the original version of the instrument and the back-translated version [20]. The overall matching between the back-translated versions with the original Spanish version indicates a high similarity between them. This supports the notion that the conciliated version of the questionnaire in Brazilian-Portuguese mirrors the original Spanish version.

Precision evaluation of the words utilized in a questionnaire is essential to avoid misinterpretation or incomprehension of the formulated questions [21]. In the present study, the outcome of the clarity/comprehension evaluation using a continuous scale was excellent (0.25; where 0 means clear and comprehensible). This indicated that the conciliated version of the questionnaire in Brazilian Portuguese language was clear and comprehensible. Importantly, the participants did not suggest re-writing of questions. These results are similar to those reported in the clarity/comprehension evaluation of the original Spanish version of the questionnaire [10]. To corroborate the clarity/comprehension data obtained in the present study, additional tests based on a three-point ordinal scale were carried out. The results of these tests corroborate that the Brazilian-Portuguese version of the questionnaire was clear and comprehensible. Additionally, the average of the Kendall’s W coefficient for the clarity/comprehension evaluation highlighted a very high agreement among the evaluations of the participants [22].

The consistency in the answers to each question of the questionnaire by the same individual was also evaluated. The questionnaire was applied twice at different moments, allowing at least one-week intervals to pass between the two applications. The consistency evaluated as the k coefficient value was 0.869, which can be considered an excellent agreement between the two applications of the questionnaire [23]. This result is similar to that reported for the original Spanish version of the instrument [10].

Online survey studies have gained attention, as the staff requirement to collect data and printing costs can be kept to a minimum. Under these bases, a pilot online survey study was carried out using the instrument generated. However, a very low response rate was reported (9.0%). In line with this, several survey-based studies, conducted using internet platforms, have reported similar and even lower response rates [24]. On the contrary, previous studies have reported high response rates (53.3 to 92.0%) using the Spanish version of the questionnaire utilized in this pilot study, but conducting the survey on the bases of face-to-face interviews in public places instead of using internet platforms [10,11,12,13]. The prevalence data generated in the present online pilot survey study should be interpreted with caution, as the response rate was quite low, and the sample was limited to Brazilian health sciences students. The same applies for the data related to the gastrointestinal and extra-intestinal symptoms reported. The main contribution of the online pilot survey study carried out in the present work is that it highlights the need to perform face-to-face interviews to successfully utilize the Brazilian-Portuguese version of the questionnaire intended to evaluate the self-reported prevalence of GRD and adherence to a GFD.

## 5. Conclusions

In this study, a questionnaire intended to estimate the prevalence of self-reported GRD and adherence to a GFD was translated to Brazilian Portuguese and tested. The questionnaire was clear, comprehensible, and generated reproducible results in the target population. The questionnaire should ideally be applied preferentially on the bases of face-to-face interviews instead of using online platforms. This strategy can help to improve the response rate and minimize bias in order to generate representative results. The present study provides an instrument to estimate the prevalence of self-reported GRD and adherence to a GFD in Brazilian Portuguese native speakers, a community that represents almost half of the South America population.

## Figures and Tables

**Table 1:** Self-reported prevalence rates.

Assessment	(+) Cases	Mean Age in Years (Range)	Prevalence by Gender (95% CI)	*p*-Value	General Prevalence (95% CI)
Adverse reaction to foods	*n* = 24 *M* = 6 *F* = 18	27 (19–47)	*M* = 33.33 (13.34–59.01) *F* = 32.14 (20.28–45.96)	0.999	32.43 (22.0–44.32)
Adverse reaction to wheat/gluten	*n* = 12 *M* = 3 *F* = 9	30 (20–47)	*M* = 16.66 (3.57–41.42) *F* = 16.07 (7.62–28.33)	0.999	16.21 (8.67–26.61)
Self-Reported Gluten sensitivity (SR-GS)	*n* = 2 *M* = 0 *F* = 2	27 (20–34)	*M* = 0 (0.0–18.53) *F* = 3.57 (0.43–12.31)	0.999	2.70 (0.32–9.42)
SR-PD Celiac disease	*n* = 0	—	—	—	—
Wheat allergy	*n* = 1 *M* = 0 *F* = 1	20 (N/D)	*M* = 0 (0.0–18.53) *F* = 1.29 (0.22–6.99)	0.999	1.35 (0.03–7.30)
NCGS	*n* = 1 *M* = 0 *F* = 1	34 (N/D)	*M* = 0 (0.0–18.53) *F* = 1.29 (0.22–6.99)	0.999	1.35 (0.03–7.30)
Adherence to GFD	*n* = 8 *M* = 1 *F* = 7	25 (20–41)	*M* = 1.29 (0.22–6.99) *F* = 9.09 (4.47–17.6)	0.671	10.81 (4.78–20.19)
Avoid wheat/gluten-containing foods	*n* = 12 *M* = 3 *F* = 9	26 (20–41)	*M* = 16.66 (3.57–41.42) *F* = 16.07 (7.62–28.33)	0.999	16.21 (8.67–26.61)

## Supplementary Materials

The following are available online at https://www.mdpi.com/1010-660X/55/9/593/s1, Supplementary Material Section S1: Questionnaire in English; Supplementary Material Section S2: Conciliated version of the questionnaire in Brazilian Portuguese; Supplementary Material Section S3: Definition and criteria of gluten-related disorders.
