# Supplementary material for: Translation, Cultural Adaptation, and Evaluation of a Brazilian Portuguese Questionnaire to Estimate the Self-Reported Prevalence of Gluten-Related Disorders and Adherence to Gluten-Free Diet
Source: Medicina (Kaunas). 2019 Sep 15;55(9):593. doi: 10.3390/medicina55090593 (PMC6780761; doi:10.3390/medicina55090593)
Supplement: Supplementary file 1 [file medicina-55-00593-s001.pdf]

Translation, Cultural Adaptation, and Evaluation of a Brazilian Portuguese Questionnaire to Estimate the Self-Reported Prevalence of Gluten-Related Disorders and Adherence to Gluten-Free Diet

S1. English Version of the Questionnaire

Please indicate with an X the answer to each of the following questions

1. Do you have some discomfort or adverse reaction when consuming wheat products?

Yes (Go to question 2, please answer only section 1)
No (Go to question 20, please answer only section 2)

ID:_________ Full Name:____________________________________Age:_______Date of birth: / / Female Male Level of education:_______________

Home Phone:_______________ Cell/mobile:________________ Email:____________________

SECTION 1

2. Have you ever been diagnosed with a disease related to the consumption of wheat or gluten?

Yes (which of the following?) No **(go to question 4)**

a) Wheat allergy
b) Dermatitis herpetiformis
c) Coeliac disease
d) Wheat or gluten ataxia
e) Sensitivity or intolerance to wheat
f) Other (Which one?)

3. Who made the diagnosis?

a) Physician or General Practitioner (GP)
b) Gastroenterologist
c) Homeopath
d) Chemist
e) Dietitian
f) Nutritionist
g) Other, who?

4. What kind of discomfort or gastrointestinal symptoms have you shown repeatedly by consuming foods with wheat or gluten?

a) Stomach pain
b) Diarrhea
c) Vomiting
d) Nausea
e) Reflux

f) Upset stomach
g) Bloating
h) Abdominal bloating
i) Constipation
j) Acidity
k) Other (Which one?)
l) None (Please go to question 7)

5. When you eat food with wheat or gluten, how often do the gastrointestinal symptoms mentioned above occur?

a) Always
b) Most of the time
c) Occasionally

6. After eating foods with wheat or gluten, how long does it take for the gastrointestinal symptoms referred to above to appear?

a) 10 min or less
b) 10 min–1 h
c) 1–2 h
d) In the first 6 h
e) Between 6 h and 24 h
f) After 24 h
g) Do not know

7. What kind of discomfort or no bowel symptoms have you had repeatedly by consuming foods with wheat or gluten?

a) Hives or rashes that itch
b) Difficulty breathing
c) Swelling of face or lips
d) Fatigue
e) Muscle pain
f) Pain in the joints
g) Lack of Wellbeing
h) Rhinitis
i) Dermatitis
j) Anxiety
k) Confusion
l) Headache
m) Numbness
n) Depression
o) Other (Which one?)
p) None **(If you choose this option, please go to question 10)**

8. When you eat foods with wheat or gluten, how often the non-intestinal discomfort or symptoms mentioned above occur?

a) Always
b) Most of the times
c) Occasionally

9. After eating foods with wheat or gluten, how long it takes for discomfort referred above to appear?

a) 10 min or less
b) 10 min–1 h
c) 1–2 h
d) In the first 6 h
e) Between 6 and 24 h
f) After 24 h
g) Do not know

10. Have you been diagnosed with any of the following?

a) Irritable bowel syndrome
b) Lactose intolerance
c) Intolerance to food other than lactose (which one?)
d) Allergy (Which one?)
e) Any type of psychiatric disorder?
f) Gastrointestinal cancer
g) Eating disorder (which one?)
h) Autoimmune disease such as diabetes mellitus.
i) Colitis
j) None of the previous diseases

11. Does anyone in your family have been diagnosed with coeliac disease?

a) Yes
b) No
c) Do not know

12. Do you keep a diet free of wheat or gluten?

a) Yes **(go to the next question)**
b) No **(go to question 15)**

13. Who instructs the diet free of wheat or gluten?

a) Nutritionist
b) Dietitian
c) Physician or General practitioner
d) Gastroenterologist
e) Chemist
f) Homeopath
g) Yourself
h) Other, who?

14. What are the main motivations for keeping a diet free of wheat or gluten?

a) Weight control
b) Having a relative with celiac disease or wheat/gluten sensitivity or intolerance
c) The gluten-free products taste better
d) A diet free of wheat or gluten is healthier
e) The symptoms triggered after gluten ingestion
f) Other reason, which one?

(Stop)

15. Do you avoid, as much as possible, wheat- or gluten-based products from your diet?

a) Yes **(Go to the next question)**
b) No **(Stop)**

16. What are the main motivations for avoiding, as much as possible, wheat or gluten from your diet?

a) The symptoms triggered after gluten ingestion
b) Weight control
c) Having a relative with celiac disease or wheat/gluten sensitivity or intolerance
d) The gluten-free products taste better
e) Avoiding wheat or gluten is healthy
f) Other reason, which one?

Thank you very much!

SECTION 2

17. Do you have some discomfort or adverse reaction when consuming a particular food?

a) Yes (Go to next question)
b) No (Please go to question 20)

18. Have you been diagnosed with any of the following diseases?

a) Lactose intolerance
b) Intolerance to food other than lactose (Which one?)
c) Any type of allergy
d) Any type of psiquiatric disorder
e) Gastrointestinal cancer
f) Eating disorder (Which one?)
g) Autoimmune disease such as diabetes mellitus.
h) Colitis
i) Irritable Bowel Syndrome
j) None of the previous diseases

19. Does anyone in your family have been diagnosed with celiac disease?

a) Yes
b) No
c) Do not know

20. Do you keep a diet free of wheat or gluten?

a) Yes (go to the next question)
b) No (go to question 23)

21. Who instructs the diet free of wheat or gluten?

a) Nutritionist
b) Dietitian
c) Physician or General practitioner
d) Gastroenterologist
e) Chemist
f) Homeopath
g) Yourself
h) Other, who?

22. What are the main motivations for keeping a diet free of wheat or gluten?

a) Weight control
b) Having a relative with celiac disease or wheat/gluten sensitivity or intolerance
c) The gluten-free products taste better
d) A diet free of wheat or gluten is healthier
e) Other reason, which one?

Stop

23. Do you avoid, as much as possible, wheat- or gluten-based products from your diet?

Yes **(go to the next question)**
No **(stop)**

24. What are the main motivations for avoiding, as much as possible, wheat or gluten from your diet?

a) Weight control
b) Having a relative with celiac disease or wheat/gluten sensitivity or intolerance
c) The gluten-free products taste better
d) Avoiding wheat or gluten is healthy
e) Other reason, which one?

Thank you very much!

S2. Brazilian Portuguese Version of the Questionnaire

1. Você tem algum tipo de mal-estar ou reação adversa quando come alimentos que contenham trigo, como aqueles que são feitos com farinha de trigo?

☐ Sim **(Vá para a pergunta 2)** ☐ Não **(Vá para a pergunta 17)**

ID:____Nome (opcional):__________________________Idade **(importante)**:____________

Feminino ☐ ☐ Masculino

Escolaridade (Último grau de estudos obtido):______________________

Contato (Opcional) E-mail: _____________________Telefone__________________________

Por favor, marque com um x a resposta para cada uma das perguntas abaixo

Seção 1

2. Você tem algum diagnóstico de alguma doença relacionada ao consumo de trigo ou glúten?

☐ Sim **(Qual das doenças abaixo)** ☐ Não **(Vá para a pergunta 4)**

☐ Alergia ao trigo ☐ Dermatite Herpetiforme ☐ Doença celíaca ☐ Ataxia por glúten ou trigo ☐ Sensibilidade ou intolerância ao trigo. ☐ Outro, qual?__________________

3. Quem deu o diagnóstico?

☐ Médico Clínico Geral ☐ Gastroenterologista ☐ Médico Homeopata ☐ Bioquímico ☐ Nutricionista ☐ Outro, quem?____

4. Que tipo de sinais ou sintomas gastrointestinais você tem apresentado de maneira repetida ao consumir alimentos com trigo ou glúten?

☐ Dor de estômago ☐ Diarreia ☐ Vômito ☐ Náuseas ☐ Refluxo

☐ Flatulência ☐ Inchaço no estômago ☐ Mal-estar abdomina ☐ Prisão de ventre

☐ Acidez ☐ Outro, qual. ☐ Nenhum **(Vá para a pergunta 7)**

5. Quando consome alimentos com trigo ou glúten, com que frequência se apresenta os sintomas gastrointestinais mencionados anteriormente?

☐ Sempre ☐ Muitas vezes ☐ Ocasionalmente

6. Depois de ter comido alimentos com trigo ou glúten, quanto tempo demora para aparecer os sintomas gastrointestinais referidos anteriormente?

☐ 10 min ou menos ☐ 10min- 1h ☐ 1-2h ☐ Nas primeiras 6 h ☐ Entre 6 e 24h
☐ Depois de 24h ☐ Não sabe

---

7. Que tipo de sinais ou sintomas não intestinais você tem apresentado de maneira repetida ao comer alimentos com trigo ou glúten?

☐ Coceira ou caroços na pele ☐ Dificuldade para respirar ☐ Inchaço no rosto ou lábios ☐ Cansaço ☐ Dor muscular ☐ Dor nas articulações ☐ Falta de bem-estar Rinite. Dermatite Ansiedade Confusão ☐ Depressão Dor de cabeça ☐ Sensação de formigamento ☐ Outro, qual?_______________ ☐

☐ Nenhum **(Se caso marque essa opção, vá para a pergunta 10).**

---

8. Quando consome alimentos com trigo ou glúten, com que frequência se apresenta os sintomas gastrointestinais mencionados anteriormente?

☐ Sempre ☐ Muitas vezes ☐ Ocasionalmente

---

9. Depois de ter comido alimentos com trigo ou glúten, quanto tempo demora para aparecer os sintomas gastrointestinais referidos anteriormente?

☐ 10min ou menos ☐ 10min- 1h ☐ 1-2h ☐ Nas primeiras 6 h ☐ Entre 6 e 24h
☐ Depois de 24h ☐ Não sabe

---

10. Você tem o diagnóstico de alguma das doenças abaixo?

☐ Síndrome do intestino irritável ☐ Intolerância a lactose
☐ Intolerância a algum outro alimento, qual? ___________________________
☐ Alergia, qual?________________ ☐ Alguma doença psiquiátrica, qual?____________
☐ Câncer gastrointestinal ☐ Transtorno de Conduta Alimentar, qual?_____________
☐ Doença autoimune como Diabetes mellitus ☐ Colite ulcerosa ☐ Nenhuma das anteriores

---

11. Alguém da sua família já foi diagnosticado com doença celíaca?

☐ Sim ☐ Não ☐ Não sabe

---

12. Você segue uma dieta livre de trigo ou glúten?

☐ Sim **(Vá para a seguinte pergunta)** ☐ Não **(pase a la pregunta 15)**

---

13. Que profissional consulta, para seguir uma dieta livre de glúten?

☐ Médico Clínico Geral ☐ Gastroenterologista ☐ Médico Homeopata ☐ Bioquímico ☐ Nutricionista ☐ Você mesmo ☐ Outro, quem?________.

---

14. Quais são as razoes que te motiva a seguir uma dieta livre de glúten?

☐ Controle de peso ☐ Tem alguém da sua família que tem doença celíaca ou intolerância ao trigo ☐ Os produtos sem glúten são mais gostosos ☐ A dieta livre de glúten é mais saudável ☐ Os sintomas que ele causam ☐ Outra razão, qual?__________. **(termina)**

---

15. Você faz o possível para não comer trigo ou glúten?

☐ Sim **(Vá para a seguinte pergunta)** ☐ Não **(termina)**

---

16. O que te motiva para fazer o possível de não comer trigo ou glúten em sua dieta?

☐ Os sintomas que ele causam ☐ Controle de peso ☐ Os produtos livres de trigo são mais gostosos ☐ Tem alguém da sua família que tem doença celíaca ou intolerância ao trigo ☐ É saudável evitar o trigo ou o glúten na dieta ☐ Otra razón, cuál?________.

¡ MUITO OBRIGADO (A) POR PARTICIPAR!

ID:____Nome (opcional):__________________________Idade **(importante**):_____________

Feminino ☐ ☐ Masculino

Escolaridade (Último grau de estudos obtido):______________________

Contato (Opcional) E-mail: ______________________Telefone___________________________

Por favor, marque com um x a resposta para cada uma das perguntas abaixo

SEÇÃO 2

17. Voce sente algum tipo de mal-estar ou reação adversa quando consome um alimento em particular que não seja feito com trigo ou farinha de trigo?

☐ Sim ☐ **Não**

18. Você tem o diagnóstico de alguma das doenças abaixo?

☐ Intolerância a lactose. ☐ Intolerância a algum outro alimento, qual?_________.

☐ Alergia qual?___________. ☐ Alguma doença psiquiátrica, qual?___________.

☐ Câncer gastrointestinal ☐ Transtorno de Conduta Alimentar, qual?___________.

☐ Diabetes mellitus ☐ Colite ulcerosa ☐ Síndrome do intestino irritável

☐ Nenhuma das anteriores

19. Alguém da sua família já foi diagnosticado com doença celíaca?

☐ Sim ☐ Não ☐ Não sabe

20. Você segue uma dieta livre de trigo ou glúten?

☐ Não **(pase a la pregunta 23)** ☐ Sim **(Vá para a seguinte pergunta)**

21. Que profissional consulta, para seguir uma dieta livre de glúten?

☐ Médico Clínico Geral ☐ Gastroenterologista ☐ Médico Homeopata ☐ Bioquímico

☐ Nutricionista ☐ Você mesmo ☐ Outro, quem?________.

22. Quais são as razoes que te motiva a seguir uma dieta livre de glúten?

☐ Controle de peso ☐ Tem alguém da sua família que tem doença celíaca ou intolerância ao trigo ☐ Os produtos sem glúten são mais gostosos ☐ A dieta livre de glúten é mais saudável ☐ Os sintomas que ele causam ☐ Outra razão, qual?__________. **(termina)**

23. Você faz o possível para não comer trigo ou glúten?

☐ **Sim (Vá para a seguinte pergunta)** ☐ **Não (termina)**

24. O que te motiva para fazer o possível de não comer trigo ou glúten em sua dieta?

☐ Os sintomas que ele causam ☐ Controle de peso ☐ Os produtos livres de trigo são mais gostosos ☐ Tem alguém da sua família que tem doença celíaca ou intolerância ao trigo ☐ É saudável evitar o trigo ou o glúten na dieta ☐ Otra razón, cuál?__________.

¡ MUITO OBRIGADO (A) POR PARTICIPAR!

S3. Figures

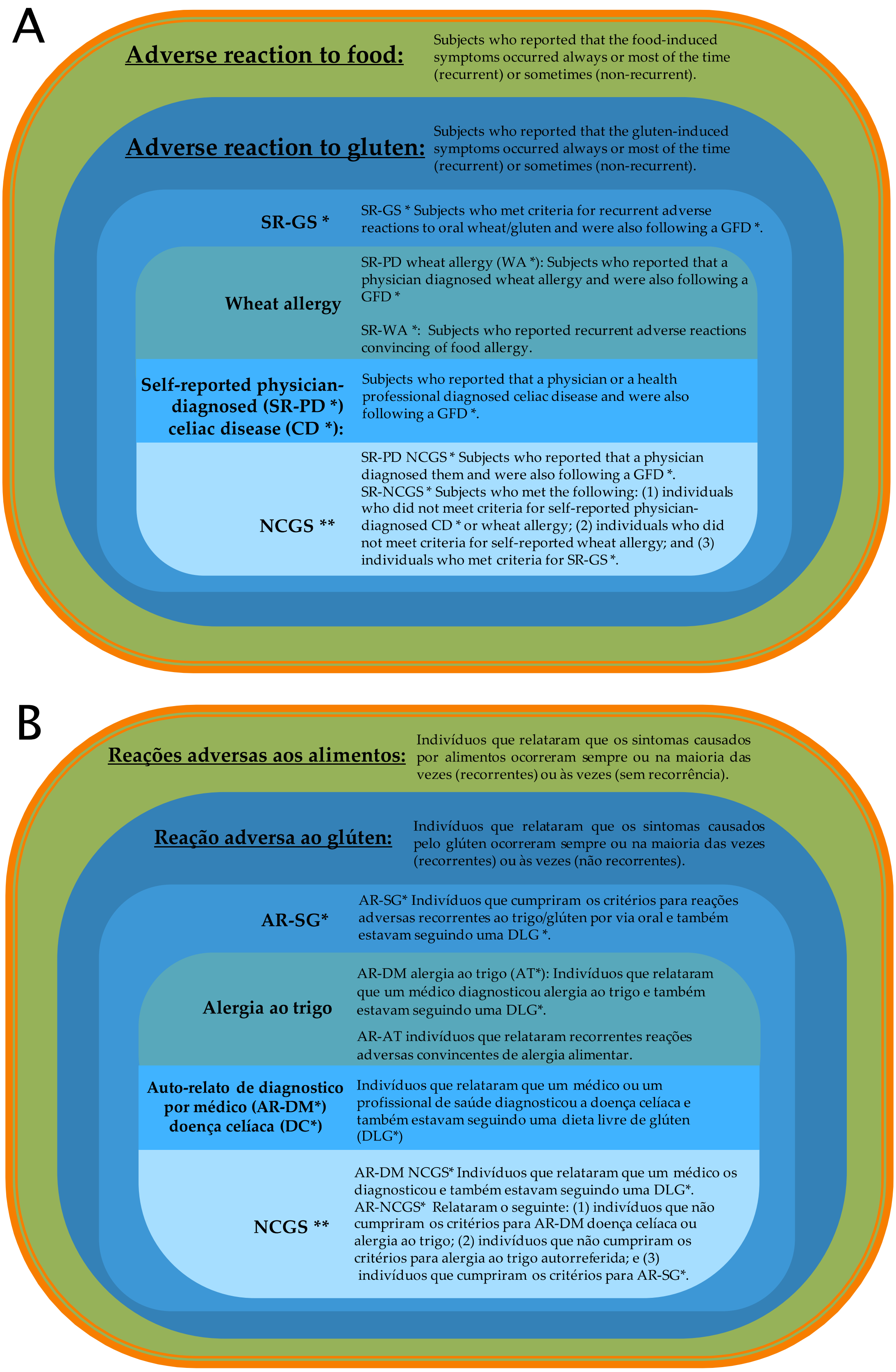

Figure S3. Definitions utilized in this study. (**A**) Definitions and acronyms in English. (**B**) Definitions and acronyms in Brazilian Portuguese. *Acronyms used: SR-PD/AR-GS: Self-reported Physician-Diagnosed; CD/DC: Celiac Disease; GDF/DLG: Gluten-Free Diet; WA/AT: Wheat Allergy; SR-WA/AR-AT: Self-Reported Wheat Allergy; SR-GS/AR-SG: Self-Reported Gluten-Sensitivity; NCGS: Non-Celiac Gluten Sensitivity; SR-NCGS/AR-NCGS; Self-Reported Non-Celiac Gluten Sensitivity. NCGS** cases reported to co-exist with WA and/or CD were excluded for prevalence estimations or classified according to the symptoms reported
